# 

**Published:** 1878-04

**Authors:** 


ESTABLISHED IN 1855.

VOLUME XVI. APRIL, 1878. NUMBER 1

116

### ATLANTA MEDICAL AND SURGICAL JOURNAL.

S.G.O. INDEXED

*MONTHLY: THREE DOLLARS A YEAR, IN ADVANCE.*

EDITOR:

J. G. WESTMORELAND, M.D.

*Professor of Materia Medica in Atlanta Medical College.*

H. H. DICKSON, PUBLISHER.

*PAX ET SCIENTIA, SED VERITAS SINE TIMORE.*

ATLANTA, GEORGIA:

*H. H. DICKSON, BOOK AND JOB PRINTER AND BOOKBINDER.*

1878.

